# Drug Addiction: Failure, Feast and Phoenix

**DOI:** 10.3390/ijerph22030370

**Published:** 2025-03-03

**Authors:** Tammy C. Ayres, Stuart Taylor

**Affiliations:** 1 School of Criminology, Sociology and Social Policy, Faculty of Social Sciences, Arts and Humanities, University of Leicester, Leicester LE1 7RH, UK; 2 Department of Social Policy and Criminology, Faculty of Arts and Social Sciences, Open University, Milton Keynes MK7 6AA, UK; stuart.taylor@open.ac.uk

**Keywords:** drug addiction, stigma, addiction, drug apartheid, jouisseaur propres, consumerism, Girard, Žižek, scapegoat, capitalism

## Abstract

This article offers a unique interdisciplinary theoretical examination of the stigmatisation of ‘drug addicts’ and its impacts on health and wellbeing. In the present conjuncture, drug addiction has become a metaphor for a ‘wasted’ life. The stigmatisation of addicts creates artificial monsters. They constitute matter out of place—addiction is dirt and the addict a form of symbolic pollution—as their excessive consumption means they are ostracised and branded as failures. Providing a tripartite framework—of failure, feast, and phoenix—this article will suggest that addiction occupies a contradictory social and conceptual space, at once cause, effect, and solution. It is in this context that the stigmatisation of addiction operates, despite the fact addicts constitute a consumer par excellence, solicited by the very system that seeks to punish, control, and cure them. Drawing on Girard’s generative scapegoat alongside the philosophical concept of the Muselmann, which parallels it, this paper will examine the hypocritical and contradictory portrayal, consumption and treatment of addiction; the social harm and stigmatisation arising from this portrayal; the systems of power and privilege that reproduce this; and how these systematically affect not only the health and wellbeing of addicts, but also their medical care and treatment. The health impacts arising from this framework will illustrate how scapegoating can lead to worsening mental and physical health, social isolation, and create barriers to treatment, which ultimately perpetuate the cycle of addiction that create public health challenges (e.g., drug-related deaths). The ensuing discussion will show how the addict is a symptom of capitalism and colonialism before it, sustaining it as well as serving as a convenient distraction from the systematic problems and illustrating the brutal realities of biopolitical power and its inherent contradictions. Only by understanding the broader socio-cultural and political implications of addiction within the context of late capitalism can we start to reduce stigma and scapegoating and focus on addiction as a medical issue rather than a moral and/or criminal one; a key to improving health outcomes.

## 1. Introduction

‘You don’t come from our world, and we don’t come from your world’ (John, a heroin addict) [1].

(p. 3)

The dominant perception among illegal drug addicts—and many of those who do not see themselves as addicts—is epitomised in the above quote, as substance use disorders (SUDs) are among the most stigmatised conditions [1,2,3,4,5], meaning that the drug addict is seen as different. Stigmatised, excluded and ‘othered’, the addict is a visual representation of what we should not be. A contemporary folk devil. A scapegoat. Someone without self-control, someone who is debauched, diseased, dirty, and inhuman [6,7,8].

Yet, whether the subject is exercise, sugar, caffeine, gaming, shopping, gambling, or social media, most of us are addicts in the broadest sense of the term. We are addicted to or dependent on particular objects or social practices or substances that sustain our identities, our relationships, and our present form of being in the world [9]. Very often, our dependence on particular consumer items or experiences is actively created and sustained by diverse cultural practices that often carry a broad range of positive connotations. Habit-forming objects and practices are widely considered an unproblematic feature of our lives together. Indeed, one might reasonably claim that habit-forming commodities have proliferated in recent years. Attempting, in some way, to capitalise upon our tendency towards repetition is now considered a legitimate business model [10]. Such incongruences are not only indicative of the dichotomies inherent in drug policy and the wider political economy (see [11]); they also illustrate capitalism’s demand to consume to excess and the dynamics of (planned) obsolescence [12]. Capitalism, of course, constantly seeks to revolutionise itself with a constant cycle of new commodities, lifestyles, and experiences, which also perpetuates the illusion of freedom and choice as well as promising to satisfy our every desire and address the constitutive lack that lies at the centre of contemporary subjectivity [13,14,15]. Problems arise if the consumption of commodities, like substances, is seen to complete the person by addressing their lack, which results in a compulsion to repeat that has the potential to lead to addiction [11,16].

Certain products, practices, and behaviours are deemed socially acceptable—desirable even. Others are framed as social ills, and this includes excessive consumption, particularly of illegal substances [11]. Severe condemnation awaits illicit drugs and their users; a malaise underpinned by illegal drugs being framed as inherently harmful, uncivilised, and resulting in addiction [17,18,19]. We acknowledge that, for some, the word addiction itself is considered stigmatising, rife with definitional issues, inconsistencies, contradictions, and underpinned by a lack of evidence [20,21]. Some even talk about the myth of addiction [22,23,24]. The word addiction is used throughout this article though as a catch-all phrase to illustrate the supposedly inevitable consequences of illegal drug use. The fairy-tale villain through which we persuade people to ‘just say no’ to avoid becoming not only addicted, but also an addict like them [25]. We purposely employ the terms addict, addicted, and addiction within this paper to reflect society’s reductionist drug discourse—as this terminology represents a crucial characteristic of how and why drugs and their users, are understood, framed, and responded to. Whilst there is a growing body of work that calls for more inclusive, less stigmatising language in relation to drugs (e.g., people who use drugs, individuals dependent on drugs, those with addiction problems), this reflects an enclave narrative spoken by a minority of academics and professionals rather than the dominant, purposeful prism of language employed to maintain the harmful status quo. We might not agree with this language, but writing a paper which challenges such structures without explicit reference to how they are maintained risks a liberal dilution of a tenacious act of wanton stigmatisation.

The addict—Jouisseaur Propres (‘ready to consummate…[their] very existence in the deadly excess of enjoyment’)—is conventionally portrayed in a double bind, as they fulfil a dual function in contemporary society [26] (p. 4). The addict is both hidden and excluded from public spaces; eliminated via environmental design and hostile architecture (e.g., the Camden bench, ultraviolet lights in public toilets) as here, they constitute matter out of place. Only non-risky civilised consumers can enjoy the rights to inclusion and personal safety provided by the state [27]. Addicts, however, are also a spectacle (e.g., in public health campaigns and the media); a public form of branding, a warning to us all that drug addiction is a demon which possesses and haunts individuals, destroying them morally and physically [6], reducing them ‘from a whole person to a tainted, discounted one’ [28] (p. 3). Drug addicts experience stigmatisation, which spoils their identities and often becomes their master status, which can be deleterious to health and increase feelings (e.g., hopelessness, alienation, guilt) that perpetuate drug use and reinforce the cycle of addiction, leading to worsened mental health outcomes [29]. Therefore, in contemporary society—as this article will show—the drug addict is a stigmatised identity. An ideologically constructed fantasy image, the addict represents a social contradiction.

## 2. Theoretical Framework

This article will illustrate how addiction/addicts represent capitalism and its inherent paradoxes, including the paradoxical condition of living, yet not living, in contemporary society [30]. Building on previous work around the stigmatisation of drug users [25], the ensuing discussion will provide an original conceptual tripartite framework—of failure, feast, and phoenix—to illustrate addiction’s contradictory place in consumer capitalism, which at once acts as cause, effect, and solution—Derrida’s [31] pharmakon and Žižek’s [32] chocolate laxative (see [16]) where the solution is built into the product itself. Drawing on Žižek, Agamben, and Girard’s theories to explain addiction, we provide a unique interdisciplinary perspective on how societal mechanisms and cultural narratives shape the (contradictory) perception and management of drug addiction, which sees the addict as a monster that needs to be punished or treated, reflecting drug policies’ contradictory twin-track approach [33]. Adopting a multidimensional approach, the ensuing discussion challenges reductionist views by providing a robust framework that enhances the theoretical understanding of addiction, thus paving the way for more comprehensive and humane approaches by recognising addicts as victims of societal dynamics and ideology. Heeding the call from Hatzenbuehler and colleagues [29] (p. 813), this article seeks to contribute to the ‘field of population health,’ which they state ‘would greatly benefit from a synthesis of…disparate literatures and from the development of a theoretical framework that provides insights into the processes that generate health inequalities among members of stigmatized groups’.

Therefore, we will show how addicts are stigmatised and how addicts constitute Girard’s [34] generative scapegoat; monstrous and inhumane, reduced to Agambian bare life, which negatively impacts their health and actively acts as a barrier to accessing treatment [3,29]. Political life (bìos) is, by definition, exclusionary, as only it can produce bare life (zoé) [35,36,37]; thus, the addict is tantamount to a failed life [13]. They are the Agambian [35] and Žižekien [36,38,39] Muselmann. The Muselmann represents the ultimate figure of abjection and dehumanisation; someone who has been reduced to a state of bare life devoid of agency, individuality, or even the desire to survive; someone who is biologically alive, but socially and politically dead. They symbolise an extreme form of biopolitical control. A failed consumer. A monstrous group that warrants social and medical control via punishment and/or treatment that enforce repressive conformity, perpetuating capitalism—and colonialism—via its system of divide and rule [11,16,17,40]; inequalities that manifest as objective violence [32], but are disavowed or méconnaissance (a wilful not knowing), if using Girard’s terms. Addicts are the scapegoats; they deflect people’s attention away from the objective violence of capitalism and the actual problems facing society [32]. Creating imaginary monsters is preferable to facing up to the truth. As such, we will show, drug addicts are the weird and the eerie [41].

Elsewhere, we have argued that the contemporary remit and nature of stigma as it pertains to drug use is shaped by consumerism and its polarisation of proficient and flawed consumption practices [25], which is theoretically advanced in this article to show how the addict (Muselmann) represents a condition of complete psychological defeat and surrender to suffering, as the addict is dehumanised and excluded, making recovery more difficult—impossible, even [3]. The term ‘stigma’ is used throughout to represent the complex attitudes, beliefs, behaviours, and structures that interact at different levels of society (i.e., individuals, groups, organisations, systems) and manifest in prejudicial attitudes about and discriminatory practices towards people with SUDs. While addiction itself is in no way antagonistic to consumer capitalism, given that addicts are in each case committed consumers beholden to the object cause of the addict’s desire, heeding capitalisms demands to repeatedly consume to excess, addicts are scapegoated, ostracised, and branded as failures in terms of their consumption choices and, de facto, their civic existence, even though the addict constitutes a consumer par excellence solicited by the very system that seeks to punish, control, and cure them [26].

Not every addict is branded a failure. The consumption of habit-forming commodities (e.g., coffee, alcohol, or prescription drugs), especially those bought from the legitimate marketplace, is often normalised and integrated into the social fabric through ideological fantasies, which are marketed and perceived as aligning with the values and demands of capitalist society. The habitual use of these commodities is not only tolerated but often celebrated as part of a successful, well-integrated, and happy life [11,15]. Only those like the drug addict are positioned outside the symbolic order of what is considered normal and acceptable in society. They use substances that supposedly subvert and do not align with the values and demands of capitalist society (although, see [11,16,42] which shows the opposite). Addicts are seen as disrupting the social order, challenging the norms of productivity and self-control that capitalism upholds and demands, leading to their scapegoating and, more often than not, their stigmatisation as part of the ideological mechanism, where society projects its anxieties and contradictions onto those who do not conform to its expectations.

‘Stigmatisation, therefore, is seen as being determined–in both form and application-by a neoliberal era of consumer capitalism, which sees engagement with consumer markets and the consumption of goods mediating all aspects of social life, including citizenship’ [25].

(p. 198)

**Figure 1:**
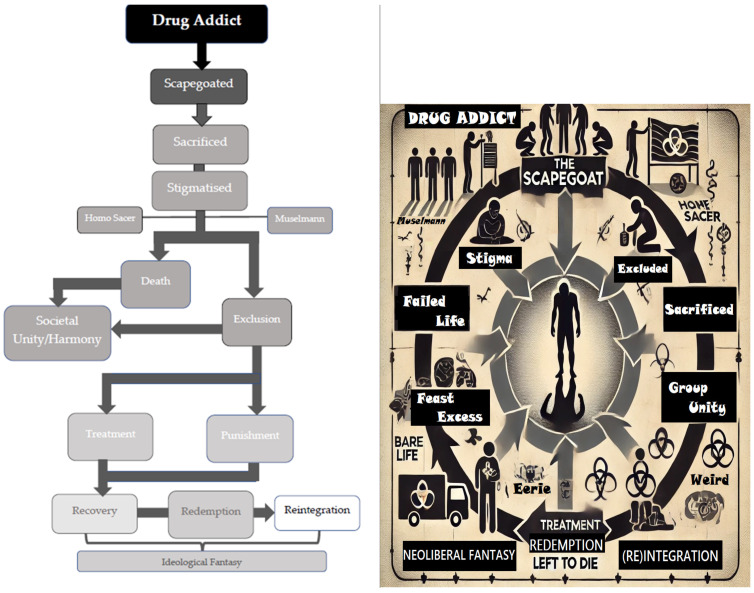
Diagrammatical representation of the theoretical framework.

Although the stigmata of scapegoats is not a given, it is symptomatic of their nefarious behaviours and signifies their difference, allowing them to be identified, excluded, and persecuted [43]. This article will show how stigma contributes to addicts’ poor health, marginalisation and treatment in society [29], as addicts are expected to seek redemption and rise like a phoenix from the ashes, when the reality is that the stigma and scapegoating make this difficult—impossible, even. It is in this context that consuming certain drugs is seen to ‘spoil’ individuals, as they are framed as transgressing social norms [5,25,44] which locks them in a cycle of addiction that creates public health challenges (e.g., the opioid crisis), as stigma is ‘a central driver of morbidity and mortality’ and ‘enables the creation of new, evolving mechanisms that ensure the reproduction of health inequalities among members of socially disadvantaged populations’ like drug addicts [29] (p. 819). As the ensuing discussion will show, however, these social norms—maintained by ideological fantasies—are contradictory, discriminatory, and harmful, as the addict is failure, feast and phoenix (see Figure 1).

Starting with how addicts are failures, living wasted lives, the first two sections of this paper show how the addict has been historically scapegoated, which has led to their stigmatisation, as addiction constitutes a failed life. This failure justifies their removal from society for consuming substances excessively (feasting); this is the focus of the next two sections, which include a discussion of how we, as consumers, feast on them. The final section discusses how addicts can redeem themselves by consuming treatment, rising like a phoenix from the ashes, although this section shows this is an illusion. The conclusion brings the preceding sections together to show how this new theoretical perspective applies in policy and practice.

## 3. Failure: Failed Life

Drug addiction has a long history of being posited, understood, and responded to on moral grounds; legacies that continue to haunt the addict today, as addiction has been both criminalised and medicalised as a result. Addiction—also known as SUDs—has been classified as an illness—a mental illness—which already implies a deviation from some clearly defined norm that can only be cured by medical intervention/treatment or, as some might proffer, control [24]. However, one might reasonably suggest that addicts are judged more harshly than those with many other mental illnesses. Perceived by the public to be responsible for their condition via bad choices, they are a burden on society and are addicted due to a lack of self-discipline and willpower [2,4,6].

Demarcating a lack of self-control, the term drug addict does not refer ‘to a bona fide patient but to stigmatised identity’ [24] (p. xvii). Consequently, the addict can be understood as a historical scapegoat [24,34]; ‘the sacrificial lamb…which unites society through the execution of violence against a common enemy’ [45] (p. 63). Throughout history society has partaken in the ritual persecution of scapegoats to create Others—Outsiders—that are demonised and portrayed as a threat to society and the dominant status quo, whose removal—either via treatment, punishment or death—is not only justified, but necessary to maintain social peace and harmonious communal living [24,34]. Scapegoating is thus an essential feature of culture and civilisation [34]; it is about divide and rule, as subsets of the population are stigmatised, oppressed, and excluded. Stigma, however, is not static. There is a hierarchy of stigma even among illicit drug users. Some illegal drugs (e.g., heroin and crack) and methods of use (e.g., intravenous use) are more stigmatising, and more embedded in racist and classist ideas of drug use than others [8,11,17]. Power operates through stigma and has been used throughout history as a form of social, or in the case of addiction, medical control, in the oppression of undesirable others, and undesirable behaviours, as capitalism, and colonialism before it, govern via a system of divide and rule [11,40].

Historically, the addict was interpellated with religion—Christianity—and the emerging biopolitics of modernity, which portrayed addicts as morally weak, mentally ill, deviant people who had become a slave to illegal/illegitimate substances; substances that took away their free will. Addiction was incorporated into the general category of insanity, feeble-mindedness, degeneracy, and/or criminality, especially among the poor and ethnic minorities that, in treatment, ‘melted into the diffuse shadow zone between madness and reason’ [46] (p. 87). These tropes were stratified along the lines of class, gender and race/ethnicity to create artificial monsters that resulted in a ‘stratification of the will’ [46], which corresponded to the stratification of industrial society itself. There was the ‘cocaine crazed negro’ impervious to bullets who went around attacking white women; the Mexican ‘Marijuana Menace’ that was linked to crime and madness, a trope also extended to Africans that smoked cannabis; and the nefarious and sinister Chinese opium dens; these were all tropes that scapegoated and stigmatised certain substances and their users, usually along xenophobic lines [11,47,48,49]. Consequently, drug taking and addiction became seen as a race and class menace; something that continues and is structurally embedded in drug prohibition today [11,17,19,47,49]. This results in certain groups of addicts enduring multiple stigmas that lead to their exclusion [5], if they were ever included.

Imbued with such overtones, the sacrifice of the addict has been justified by the mythologies created to conceal the truth. This is true whether referring to the colonial imaginary (see [17]) or the crystal meth imaginary see [50]; both provide a sense of stability and wholeness, concealing the fragmented nature of reality and the self, as the imaginary serves to mask the inconsistencies and gaps that exist in our experience and understanding, often concealing the underlying power structures and their inherent contradictions. These myths are built around the scapegoat mechanism [34,51]. The myths of the imaginary become more real than reality itself, making them seem natural and unquestionable ‘while fetishistically disavowing the harms arising from the system itself and its (legal) products, including its objective violence’ [11] (p. 26). As illustrated elsewhere, only controlled consumption of ‘substances of the colony’ and, later, capitalism, occupy a legal, privileged position (e.g., tobacco, alcohol, sugar), while everything else is controlled and criminalised (e.g., opium, cocaine, cannabis), particularly substances that are seen as coming from other often barbaric, uncivilised orientalist cultures [17], provided by foreigners that want to ‘ruin our way of life by corrupting it with their own peculiar enjoyment’ [52] (p. 22). Instead, the control and regulation of substances is actually tied to the civilising process and is based on notions of morality, religion, race and class; some substances were moralised and seen as a vice, while others were not, and this also included modes of consumption—medical versus non-medical, controlled versus uncontrolled/addicted and legitimate versus illegitimate [11,18,53]. Only non-medical, uncontrolled/addicted and illegitimate consumption practices were said ‘to lead to slavery, as manifested in addiction, and to ruin, as manifested in disease, pauperism, and depravity’ [53] (p. 125), as addicts become a fundamentally different entity just by consuming the wrong—unprivileged—substances [19]. Addicts thus constitute the weird and the eerie; fundamentally out of place in society, they do not belong, and they disrupt the social order and evoke a deep sense of societal unease [41], as capitalism’s ideological fantasies create a semblance of normality [54]. As a result, drugs are blamed, scapegoated, and stigmatised to prioritise the exigencies of capitalism and before that, colonialism, and its substances, as addiction is seen as incompatible with and undermining labour and production integral to (industrial) capitalism at the time [17]. Addiction has been linked with poverty, disease—of the body and mind—and death; historical legacies that continue today and justify the sacrifice of the addict as the use of and addiction to illicit drugs takes profits away from the legitimate market’s substances (e.g., alcohol and laudanum) and capital’s pursuit of profit [11,17,43,47].

Resultantly, addiction is seen as a threat—a threat which arises due to poor individual consumption choices. In the neoliberal ethos, addiction is seen as an individual phenomenon. Medical discourse and biopolitics treats addiction as being in and of the individual—situated in Western ideals of self-control, weakness, productivity, and individualism [55]. Yet, consumption is no longer a rational choice, but a compulsion [13] and the consumption of drugs is no different. Drug addiction, therefore, is seen as the inevitable outcome of a lack of individual control and a lack of individual responsibility, firstly through the decision to consume a singular, inappropriate commodity, and secondly through the inability to manage this in a way that is acceptable. As such, the addict only has themselves to blame! Whilst addiction is therefore a failing, it is the wider connotations attached to the stereotypical addict that cement their position as a social failure via an inability to work, ill health, failure to parent, reliance on welfare, committal of economic–compulsive property crime and their ability to destroy communities, neighbourhoods and town centres, despite the reality.

## 4. Failure: Failed Consumer/Consumption

Addicts’ erroneous consumption of drugs becomes the defining factor in their lives [25], referred to as their master status [56]. Hence, the addicted become the collateral casualties of consumerism, ‘portrayed as lax, sinful and devoid of moral standards’ [13] (p. 34). Drug addiction has become a metaphor for a failed and wasted life, as addicts constitute ‘human waste’ [13,57]. This reflects a ‘troubling symbolism’ which ‘threads through the moral ground of self-abandonment to both addictive substances and state help’ [58] (p. 99) as the ‘oppression of prohibitive power is replaced by benevolent administration of Foucauldian biopolitical control and regulation’ [39] (p. 113). Those who encounter addiction, or more precisely those who encounter addiction who are drawn from certain populations, are framed as a menace to the status quo—an already marginalised population, who, despite the freedom offered by contemporary consumerist society, are unable to engage with, or contribute to it ‘properly’, and who therefore endanger and threaten to pollute our way of life [57]. As a result, they are framed as a problem to be managed via scapegoating and stigmatisation, ‘legitimising continued adherence to polices of drug prohibition and the bifurcated criminal justice system’s coercive/punitive response to those deemed problematic drug users [25] (p. 205)—a process which in itself sees those addicted to drugs become an essential commodity to the criminal justice and prison–industrial complex. The addicts, like the substances, however, are what we refer to here as abject objects of ideology. Here, the ritual sacrifice of the drug addict is a foundational act; embedding violence in the cultural order that is justified and necessary to reinforce social cohesion, moral boundaries, and which perpetuate the ideological fantasies that compel us to desire products, lifestyles, substances and identities that align with the values and interests of consumer capitalism, or risk becoming failed consumers. By labelling some lives as failed, these individuals—drug addicts—become the generative scapegoats [34].

The drug addict is blamed for the destruction of families, communities, crime, poverty, immorality, and disease, as society projects its fears, anxieties, and moral judgments onto addicts, and these projections are legitimised in drug policy and legislation [25,43,59,60,61], justifying their exclusion and/or sacrifice (e.g., in countries like Singapore, possession of even small amounts of certain drugs can lead to the death penalty), in order to restore social harmony, unifying the non-addicted majority against a common ‘enemy’, reinforcing social cohesion and moral boundaries [34]. As such, drug addicts are framed as the problem, which distracts from the underlying issues—poverty, mental health, discrimination, trauma, and social inequality—that are often the root causes of addiction. The addict becomes pharmakos—the poison and the cure—a human scapegoat used by the state in certain rituals [34]. The heroin epidemic of the 1980s and its links with crime and HIV/AIDS detracted from the recession, socio-economic austerity, poverty, and high rates of unemployment plaguing society at the time, while the current opioid crisis in America has detracted from the unscrupulous behaviour of the pharmaceutical industry, doctors and politicians. The drug addict—pharmakos—is chosen to be expelled or sacrificed during times of crisis to cleanse or purify the community, as they are held responsible, thus maintaining social cohesion. Only via their removal—by treatment, punishment or death—can harmony, order, and happiness be restored. The scapegoated drug addict is thus both the cause of the disorder and the means of resolving it, much like the dual nature of the pharmakos [31,34], as humans constantly evaluate each other’s actions, drawing near by imitation while simultaneously justifying their distance (difference) by scapegoating the drug addict to mark their own identity and enhance societal solidarity and group unity.

In an epoch of fear, the addicted have been framed as a threat that needs to be managed. This belief has justified the extension of prohibitive drug laws, the application of these to drug users, and the implementation of ever more punitive responses to such individuals, despite the lack of underpinning evidence [11,17,19]. Addiction discourse is moralising and normative [46]. Although dependence has no place in contemporary society (e.g., a dependence on welfare; substances; housing, healthcare, or other state institutions is discouraged), sharing consumer dependency ‘is the condition of sine qua non of all individual freedom’ [62] (p. 84). In fact, addiction is a convenient term to describe disapproved consumption patterns, which acts as a form of consumer manipulation, but could be applied to other disapproved behaviours and consumption practices. Consequently, drug addiction is a metaphor for a failed life, which has deteriorated/degenerated under neoliberal consumer capitalism and its monopolisation and mobilisation of desire, as capitalism exploits the metonymy of desire—desire is always a desire for something else, something better—creating a never-ending process of continual deferment and dissatisfaction, which is exploited by capitalism, as it keeps us buying and consuming [14,63,64], as people are trapped in a culture of liquid consumption where individual aspirations are formed by observing others.

Driven by mimetic desire, societies create a class of failures—the drug addict—who is blamed for their inability to succeed in a liquid modern world [43,62]. Therefore, addiction can be viewed as a mimetic cycle where individuals imitate behaviours and use substances that are seen as desirable (e.g., champagne), particularly to others. Whether it is imitating the rituals around smoking and appreciating cannabis [56] or heroin [65], drug use and addiction, for some, provide structure, meaning, purpose, prestige and a sense of community/family, as drug users—think they—desire what the others have/desire (see also [66,67,68]). This is because our desires are never our own, as everyone desires and imitates others’ desires, which has the potential to create jealousy, rivalry and eventually scapegoating, stigmatisation and violence as everyone desires the same things and everyone starts to resemble everyone else [43]. The addict, however, is also their mimetic rival, a community of scapegoats that act in this instance as a model of what not to be, but also ‘dangerous, deceitful, unreliable, unpredictable’, a threat to them and society, especially if they suffer from multiple stigmas (e.g., poor, homeless, ethnic minority) [5] (p. 8). In the UK, homeless spice (synthetic cannabinoid) users have been exiled from public (consumption) spaces to areas described as being ‘so unsafe…now’ [69] (p. 3). In Kensington, Philadelphia, the congregation of excluded opioid users has left residents afraid to walk down the street [1,70], but they have also become a spectacle, a source of entertainment, as users are filmed when they are in intoxicated, zombie-like physical states for people’s amusement on social media [71].

Therefore, shame and stigma are unequally distributed across the same consumption practices, often of the same substances [72], as the destructive nature of illegal drugs and addiction is emphasised among already marginalised groups. The shame and stigma attached to these groups helps to maintain the social order by reinforcing the dominance of the majority group. The unequal distribution of stigma illustrates ‘the distinction between those who are included in the legal order and homo sacer’, which ‘is not simply horizontal…but is increasingly also a vertical distinction between two (superimposed) ways in which the same people can be treated’ [39] (p. 124). Substances have been used to teach universal morality to the masses, promoting the ideal of good citizenship by the ethical elite. Therefore, the scapegoating of addicts serves to detract from the harms arising from the political economy, drug prohibition, and the privileged substances of capital, as well as relieving the tension we derive from our inability to desire directly, always desiring what the other desires as we seek to turn ourselves into the object of the Other’s desire, which can only be achieved through consumption of the right substances, services, and lifestyles, which have had the dangers removed as part of the biopolitical agenda [39]. It is in this context that the solution to all of our problems and desires is sold to us in the legitimate commodified marketplace. Products, substances, and services offer us quick-fix solutions, which promise to assuage our anxieties, despite being rife with ambiguity and harm [11,15,42]. Here, the problem is often sold as the solution, and (excessive) consumption and enjoyment solicited. The consumer, however, is forever cheated by its deferred promise of fulfilment that results in dissatisfaction, which perpetuates further desire and consumption, not only driving capitalism forward but often resulting in excessive (binge) consumption and addiction [15,16,40].

## 5. Feast: Excessive Consumption

Consumer capitalism relies on excess, as ‘excess drives the economy’ [52] (p. 27), creating a logic of erratic excess [32,38]. Consuming to excess is an ideological imperative. The ‘morality of capitalism’ is ‘too much is never enough’ [73] (p. 240–241), as people seek more, more, more [63]. Meanwhile, addiction is an evil excess threatening the stable social order, as today’s biopolitics fights against the dangers of unrestrained excessive consumption [39]. The addict consumes to excess, thus making them—prima facia—the perfect capitalist subject—the hyperconsumer [74].

Capitalism pushes consumers beyond fulfilling basic needs and encourages the pursuit of excess, often influenced by advertising and the creation of ‘ever new perverse and excessive desires,’ [36] (p. 63) which addicts heed capitalism’s demand to enjoy. Like all humans, addicts are subjects of desire ‘possessed by a strange drive to enjoy life in excess passionately attached to a surplus which sticks out and derails the ordinary run of things’ [64] (p. 499); addicts attempt to capture the surplus of pleasure that the symbolic order typically represses. It is ‘the paradoxes of surplus enjoyment that sustain the topsy-turviness of our time’ [75] (p. 2). While some addictions are encouraged, celebrated even, as a normal part of capitalism (e.g., the entrepreneur is addicted to working hard and making money), others are stigmatised (e.g., drug addiction). The drug addict is a paradox. On one hand, the problem with the drug addict is they take control of their own pleasure and enjoyment, bypassing capitalism and its symbolic order, pursuing a purely autistic jouissance that implodes the desire to consume other commodities [36,76], meaning it is not welcome in consumer society. The addict’s experience becomes centred around the immediate gratification provided by the substance, rather than the mediated desires structured by the symbolic order, as addiction provides an obscene jouissance.

Addicts pursue a pleasure beyond that which is good for them, often leading to self-destructive excesses (see [11]). Here, the addict desires ‘an excess of obscene life’, which is what makes life worth living in contemporary society, as there is pleasure in displeasure [36] (p. 182). However, this is an excessive pleasure that the conscious subject and society (the symbolic order) cannot tolerate, as we are expected to forgo excessive and destructive pleasure in the interests of individual and public good [40]. Everything in moderation. The addict contravenes the acceptable and commodified versions of enjoyment tolerated by society, ignoring the restrictions and limitations the symbolic order puts on enjoyment as the addict consumes to excess. Consequently, addiction can be understood as an escape from the symbolic deadlock created by the demands and contradictions of capitalism as the addict relentlessly pursues a jouissance outside the symbolic limits, heeding capitalism’s command to enjoy!

In contemporary capitalism, addiction is, subsequently, a symptom [76], as addicts use drugs to cope with the underlying discontent and emptiness that characterises capitalist societies, as they attempt to fill the void left by the lack of genuine meaning and connection in people’s lives. Like consumerism, addiction is also a sinthome, as it organises the subject’s enjoyment and relationship to the symbolic order through which individuals navigate the pressures and inconsistencies of society [39], finding in substance use and addiction a paradoxical form of stability, identity and belonging. It is through the sinthome that the subject’s fundamental fantasy is articulated and maintained [77]. Addiction, in this case, is not just a symptom to be interpreted but a fundamental part of how the individual experiences and manages their existence. Contemporary capitalism has fostered a culture of instant gratification and passive consumption, which can prevent more meaningful and fulfilling engagements with life [54,73]. Addiction can provide meaning (structure, identity and purpose) and serve as a stabilising function, helping people to manage and navigate the inconsistencies, uncertainties, anxieties, and pressures characteristic of life in competitive neoliberal society [11,16]. The addict also obeys capitalism’s demands to enjoy and consume to excess, as addiction is depressive hedonia [73] (p. 22)—‘an inability to do anything else except pursue pleasure’ that can paradoxically yield very little pleasure for the addict bar momentary relief. It is not just the pleasure obtained from the drugs; addicts also find pleasure in chasing the high, transgressing the law, and using substances to stave off and avoid the pains of life and of withdrawal, as addicts repetitively use substances no matter how painful or pleasurable, they are, thus trapping the addict in a cycle of addiction (see [11]).

The addict becomes trapped in these traumatic (symbolic) repetitions in their repetitive circuit of jouissance. The more the addict obeys the demands to enjoy, however, the more guilt, shame and anxiety they feel, which further perpetuates consumption of drugs [11], as addicts get ‘caught in the endless repetitive cycle of wandering around in guilt and pain’ [36] (p. 62). The addict becomes society’s scapegoat [43], which results in them being ‘eliminated from the world of the living’ [78] (p. 280); addicts become the ‘undead’ [36] (p. 62). The mechanisms of biopolitics have achieved their goal of absolute control, thus rendering the addict incapable of meaningful existence (unless they buy redemption), which leads to an existential void where traditional forms of political and social engagement become impossible and the addict becomes homo sacer; society’s scapegoat [43].

In an extreme form of dehumanisation, scapegoating places the addict so far beyond the boundaries of normal human existence that it constitutes an extreme form of othering; the Muselmann. Reduced to mere biological entities, stripped of their humanity, dignity, individuality, and agency, the drug addict epitomises the ultimate extent of biopolitical control, where state mechanisms exert such absolute power over these individuals that their basic humanity is obliterated, rendering addicts incapable of meaningful existence [38]. This state can be seen as eerie in Fisher’s [41] sense because it embodies a profound disconnection from the usual human experiences arising from the existential void and the complete negation of life’s normal rhythms and meanings. Thus, the drug addict is biologically alive but symbolically dead [11]. The reduction of the drug addict to a mere biological entity creates a sense of the bizarre and the out-of-place [57], which can also be considered the weird [41]. It represents an extreme form of power that has total control over life and death. In the UK, this has recently been demonstrated in the record numbers of drug-related deaths. Very little has been done by governments and policy makers to effectively reduce drug-related deaths in what Stevens [79] has referred to as a moral sidestep. This moral sidestep justifies ignoring the evidence base that exists, which may save their lives, and instead sees such deaths as acceptable, even socially beneficial; after all, it is the most socio-economically deprived, those that are a drain on society, that are dying anyway. Like the poor slum dweller, the addict, is ‘homo sacer, the systemically generated living dead of global capitalism’ [80] (p. 425).

Addicts are subsequently stigmatised and scapegoated, as they do not adhere to societal expectations or exhibit the self-control demanded by society and embodied in its products/consumables and campaigns (e.g., Drink Responsibly). Society bribes its consumers to be obedient via stigma and scapegoating, which sets the criteria for their inclusion and exclusion [37]. Addiction therefore contravenes the acceptable and commodified versions of enjoyment tolerated by society and ignores the restrictions and limitations the symbolic order puts on enjoyment. As a result, the ensuing persecution, stigma, and exclusion is an attempt to shape and control consumption practices, acting as a warning to us all of what happens if we do not obey; we will be sacrificed. The sacrifice of the addict is thus justified [43]. However, the addict, dependent on substances for the purpose of this article—though they could just as easily be addicted to sex or gambling—that consumes to excess, which is unwelcomed in society, despite being solicited by the very system that seeks to punish and control it, as the logic of erratic excess reveals the contradictions inherent in capitalism [26,38]. So, whilst fast food is readily available, we must eat responsibly and not become obese; whilst welfare payments are available to those not in work, recipients should spend these responsibly and not buy superfluous items such as cigarettes or alcohol. Our ability to consume, and to consume in the correct way, is therefore indicative of our social status—buy things, but only the right things, spend and incur debt but pay your bills, enjoy but not too much, have fun but do so safely [11,16]. In the UK, free market access to alcohol and online betting is accompanied by the contradictory messages to drink and gamble responsibly, as pleasure is diluted by the contradictory logic of sacrifice and capital [26].

The simplistic narrative of addiction which predominates media representations tapers a complex social issue into a straightforward personal (moral) failing [6,19]. Whilst such depictions have long been a mainstay of drug-related educational campaigns, news media and film industry narratives, the emergence of lifestyle, fly on the wall, and reality TV programmes have further expanded our ability to peer into the lives of the addicted and to intrinsically link these to wider failures of citizenship [81]. In doing so, they allow ‘the bemused gratification and self-assurance of mass audiences who can revel in the certainty that they are not like those shown on screen’ [58] (p. 107). Serving as a Durkheimian tool of functionality, this allows ‘society to appear as a separate and somewhat immaculate entity’ [82] (p. 1), assuring viewers that we are better than them as we use more acceptable drugs and consume in more morally acceptable ways—we are responsible consumers; they are not. These representations have created a hyperreality around drugs and drug addiction that has become more real than reality itself [6,83,84]. A false reality created to distract people from the veracity of social life,) diverting the public’s attention away from wider and more important socio-political issues; ‘the spectacle [of the addict] has become a permanent opium war’ [83] (p. 44). Blaming the addict for many of society’s problems temporarily restores social harmony and detracts from the real issues, preventing individuals from recognising the true source of their issues (e.g., structural inequalities and internal contradictions), namely the objective violence that is embedded in the very structure of society and its systems [32]. As Buchanan [85] highlighted, ‘the causes and solutions to problem drug use are much more to do with socio-economic factors than physiological or psychological factors’ (p. 387).

## 6. Feast: Consumption of the Addict

Yet within the landscape of consumerism, these ‘burning effigies’ [13] (p. 39) of the addicted are also entwined with aspects of pleasure and consumerism. Not only can one feel good about oneself through engagement with media products, one can enjoy the banquet of judgemental and morally masochistic insights, which allow us to feast on such failed lifestyles. There is a plethora of TV ‘reality’ shows (*Celebrity Rehab with Dr. Drew*, *Recovery Road*, *Real Housewives of Recovery*, and *Intervention*) depicting the stereotypical ‘spectacle’ of the addict that society consumes alongside a new phenomenon called ‘tranq tourism’ [71]. Tranq tourism is a consequence of the opiate crisis in places like Kensington, Philadelphia, that has been the subject of documentaries and viral videos uploaded to social media platforms like Youtube and TikTok. There are now ‘over 150 channels dedicated to Kensington and all the things that take place’ there, as social media influencers make money off the back of human suffering, since watching intoxicated addicts has become a new form of dark voyeuristic entertainment [71]. The image of the addict has little material reality or physical presence, but a hugely affective consequence.

Such programmes and videos provide nebulous definitions of addiction whilst perpetuating fallacious conceptualisations of its causes and effective treatment responses [86]. Many will consume these to excess as binge-watching—alongside other forms of binge-consuming (e.g., binge-eating, binge-drinking)—characterise contemporary capitalism, depicting a form of excessive and greedy consumption that often results in addiction. Despite living in a binge culture, binge-consuming is also considered troubling from a public health perspective and merges moral management and personal health, as addicts pose a biopolitical threat. Here, stigmatisation acts as a performative biopolitical process of power ‘that constitutes the very conditions under which legitimate subjects emerge’ [87] (p. 194). However, in today’s society of the spectacle, the image of the addict—the one replicated and maintained in popular culture—takes precedence over the reality. Whether it is the no-good junkie on the streets/in prison, or the celebrity addict living the high life, addiction sells. As Ayres and Taylor [25] contend, Russell Brand has made a living from his addictions, whilst Kate Moss’ career skyrocketed after being dubbed ‘Cocaine Kate’ with her ‘Rehab’ story donning the pages in the high-class glossy magazines.

The subjects’ position within the symbolic order is mediated by the fantasies we are all forced to create. In this sense, reality television is merely ‘the extension of disavowed cynicism of commodity fetishism to wider, previously non-commercial social discourse’ [88] (p. 88). Instead, we vicariously and voyeuristically enjoy these societal spectacles, which are packaged up and sold to us as enter/infotainment. These spectacles are carnivalesque, as they depict a ‘ritualised mediation between order and disorder’ [89] (p. 32). It is in this context that we see the blurring of reality and fiction in the society of the spectacle [83]. Instead, a hyperreality has been created around addiction, based on limited samples of specific populations [6,11,25]. It is this hyperreality surrounding the addict that has influenced thought, behaviour, and human action more than actuality [19]. We know very little about functioning addicts and ignore the fact that most of us are addicted to something, whether that be sugar, caffeine, shopping or exercise. Instead, the simulacrum of addiction has driven the hyperreality, believed and experienced by those feasting on the image, symbolising the murder of reality [84]. Like the substances themselves [11], the media and popular culture provide a reality deprived of its substance, and like other products in the contemporary marketplace, they also function as sublime objects of ideology via the process of desublimation [37,54,77], as people can also buy their own redemption through consumption.

## 7. Phoenix: Penance and Redemption Through Consumption

Addiction is an artificial problem that has been created so that the marketplace can provide immediate solutions to the imaginary problem. Redemption from addiction is therefore possible, but only if bought through the legitimate marketplace, as drug prohibition, alongside the punishment and treatment of the addict, is big business. Redemption through consumption occurs on two levels in contemporary society. Firstly, the marketplace proffers quick fix solutions to every aspect of life, including addiction. Secondly, people can also buy redemption from being nothing more than an egotistical consumer, as the ‘anti-consumerist sentiment’ to do something is incorporated into the very thing being consumed [26]. Importantly, cultural constructions of addiction indicate a proclivity towards two beliefs; that it is easy to ‘get clean’—you just have to engage with ‘rehab’; and that the most effective rehab pathway is via private industry rather than state-funded services [86], to the point that certain media mediums act as ‘a form of product placement and advertising for private practice’ [81] (p. 21). Indeed, the field of addiction, as in every other consumer market, has recognisable brands—to the point that when a celebrity seeks treatment in the UK (a prominent theme in news media reporting of addiction see [44]) they are said to be ‘entering The Priory’ rather than drug treatment per se. The brand is so established that there is no need to explain it—it is akin to ‘going for a McDonalds’—a fact not lost on celebrities’ public relations teams with this representing the first step in managing their public redemption.

Brands that denote wealth, status, and luxury—such as entering The Priory—become a form of conspicuous consumption, which command exorbitant prices, as people are buying more than just a commodity as drug treatment represents a particular lifestyle; a cultural experience that is not attributable to its intrinsic value, but because it is desired by others (e.g., celebrities) [26,43,54]. The descriptions of drug treatment facilities sound more like luxurious holiday experiences/locations than rehab (e.g., websites state: ‘facility is both luxurious and homey…beach cabins with all the latest amenities and creature comforts…all our rooms: top-of-the-line linens; Wi-Fi, flat-screen televisions with cable, satellite, and DVR…organic food prepared by fully trained chefs,’ and ‘a striking Grade I listed building, overlooking beautiful parklands’). Luxury sells and cost does not matter as credit is available, encouraged even, fostering ‘the illusion of classlessness’, as it indicates a higher-class identity [90] (p. 575) for the repenting addict. In contemporary society, ‘living on credit, in debt with no savings is the right and proper’ way to live [13] (p. 80), as the commodification of experiences is prioritised in contemporary cultural capitalism [26]. Drug treatment centres portray and sell healthy luxurious—often ecological—experiences/lifestyles (e.g., ‘organic food and beautiful parklands’), which are necessary if the addict is ever to find happiness in the future. They are offered a ‘deceitful promise of future satisfaction’ and happiness that can only be achieved via ‘sacrifice and renunciations’ [91] (p. 154–155). Addicts can seek redemption by entering drug treatment—but only the right drug treatment—which they consume to make their lives meaningful, to redeem themselves, to achieve a quality of life that facilitates the authentic fulfilment of their true selves and adheres to one of cultural capitalism’s commodified/proffered lifestyles [26] as the market ‘manipulates the desire to desire ever new objects…modes of pleasure’ [64] (p. 496–497).

The treatment industry itself—alongside recovery and abstinence—has become a new source for the manipulation and control of enjoyment, and thus a substitute for addiction [76]. As Marx said ‘liberation is possible but only through Capitalism’ [92] (p. 160) and engaging with drug treatment services promises the addict something better; a better tomorrow, but only if they consume the treatment on offer. As the stigmatised other, marginalised, dehumanised and scapegoated, addicts need the right sort of treatment if they are ever to be reintegrated into society and obtain happiness and fulfilment [34]. Ironically, stigma acts as a barrier to treatment and healthcare, especially publicly available treatment services where the treatment of addicts reflects their flawed, failed and diseased status as the medical profession, like other agents of the state (e.g., police, courts) enact the stigma, which negatively impacts on treatment outcomes that is exacerbated by the moral model of SUDs/addiction [3,5,93,94,95]. Interestingly, people with SUDs/addiction are stigmatised and perceived more negatively than people with other mental illnesses as they are seen as more unpredictable, more dangerous, less able to make decisions, and more responsible for their addiction, illustrating the stereotypical perception of the addict, as addiction continues to be viewed as a moral failing and personal weakness [2,3,4,96]. Also, addiction treatment is often separate from mainstream healthcare, even when it is not private, provided by specialist services employing specialist workers, further marking addicts out as the other and further excluding them from the mainstream [95]. Private treatment however, proffers a better experience; after all, in today’s society, the consumer is constantly told that you get what you pay for! You cannot put a price on happiness! Thus, the relentless search for happiness means once the addict enters treatment and desists from substance use, the addict’s pursuit of happiness ‘will be continued at the level of the relationship with the institution’ and its staff [76] (p. 251).

Like everything else, there is an excess of different treatment options and treatment providers on offer that perpetuate the illusion of freedom and choice, but actually invoke anxiety, stress, and inertia in case the wrong choice is made as people strive to keep up with the ever-changing demands and expectations of capitalist society and its erratic excess [13,36,62]. As Horkheimer and Adorno [97] noted long ago, the diner must choose from the menu. Our choices are prescribed. We can choose only from those options presented to us, and vast swathes of life are entirely absent of the supposed positivity of choice. We cannot choose to disempower the oligarchy. We cannot choose a fundamentally different political economy. We can only choose from the array of options available in the legitimate marketplace or risk the consequences (e.g., exclusion and criminalisation). Faced with an array of treatment options, addicts can deal with the underlying symptoms (e.g., trauma)—that may invoke a sense of the weird (see [41,73])—and/or access services that help the individual manage their addiction in a way that is less harmful (e.g., clean needles), which includes substitute prescribing that constitutes/illustrates Derrida’s [31] pharmakon. Hypocritically, drug treatment will often substitute illicit drugs for the same pharmacological substances provided by the capitalist market and big pharma (e.g., Glaxo Smith Klein), that can be equally harmful and/or habit-forming, but generate huge amounts of revenue for the capitalist market. Globally, the market for treating addiction was valued at USD 8.28 billion in 2022, and is predicted to increase by 6.4% to USD 14.47 billion by 2030 [98] as habit-forming products proliferate further, the world becomes more addictive [10], and treatment is prescribed as the only solution to the problem. Drug treatment promises a hedonic model of health and happiness upon recovery in accordance with society’s reductive model of health [73].

Whether you are a celebrity or not, on exiting treatment, newspaper headlines quote that ‘rehab saved my life’, demonstrating how an addicted lifestyle is not worth living, but also how the addict’s life and moral compass has been reconfigured through treatment, allowing them to rise phoenix-like from the treatment ashes, having attained salvation through the same consumer marketplace they failed to adequately navigate in the first instance. In fact, such salvation can lead to the once fallen becoming an attractive commodity, once again having added a lawless yet redemptive quality to their marketability [96]. Drug treatment, therefore, is not solely about recovering from addiction. It is about recovering one’s place as a citizen and re-joining society as a responsible and moral consumer, as the life of an addict is a life lost. Yet this can be remedied—with life given a renewed purpose—through recognisable brands, which symbolically indicate (regardless of the ultimate outcome of such treatment) that the individual has finally made a responsible consumptive choice, whilst also emphasising individual hard work and discipline alongside being in the ‘right’ treatment centre with the right staff and the right service. Crucially, this sits against a backdrop of reduced funding for public drug services in the UK, placing them at risk of being dismantled [99], meaning that treatment opportunities for the majority have been limited and are lessening, with an increase in the number of drug users not engaged with treatment services [92]. Despite governmental promises of increased funding [33], it is questionable whether those services, which have all but disappeared, will automatically return [100]. It is likely that they will not, as stigma also reduces the willingness of policy makers to allocate resources [101], let alone the resources needed to provide more compassionate and integrative drug treatment that reduce stigma and promote inclusion.

For the majority, therefore, penance is either out of reach or comes at a price, demonstrating how the addiction treatment market is worth billions, despite the inconsistencies, lack of evidence base, and there being no agreed-upon definition of addiction or recovery [3,21,22,102]. Instead, it is used as an ideology to explain the danger and inferiority of the addict. Only via their expulsion, death, or redemption can societal order be restored. The addict unites societal fears (e.g., crime, poverty, violence, insecurity and disease) within a single person/group of people —disavowing the reality that many of these fears are a by-product of and/or inherent to capitalism. It promises that if addicts are removed from society and cured then these fears/issues would be resolved. It therefore creates, maintains, and reinforces the social and racial hierarchies integral to the ideological machinery, which detracts from the objective violence of consumer capitalism, keeping the ideological fantasy alive [11,16,32]. Ideological fantasy constructs the blameworthy other as the drug addict, whose fantasy image justifies the addict being the ideological scapegoat.

Therefore, the burgeoning private treatment market is forecasted to flourish, as ‘the world will get more addictive in the next forty-years’ [10] (p. 18), particularly if ‘addiction treatment manufacturers and buyers of treatment items are concentrating on bringing issues to light among individuals and medication storekeepers about ill-effects of medication misuse and significance of addiction treatment to build the client pool’ [103] (p. 2). The US opiate crisis is a good example of the moral maze of addiction and consumerism. Despite initially arising from doctors overprescribing of opiates—illustrating Derrida’s [31] pharmakon—because they were being incentivised by the pharmaceutical companies, or acknowledging that the opioid crisis might be a response to the pressures and harmful subjectivities arising from neoliberal capitalism [11,104], this is disavowed. Alternatively, the fate awaiting opioid addicts illustrates their flawed consumer status, as their citizenship is suspended—they are reduced to bare life—via a process of othering, with one US state introducing a three-strikes-style policy for people who repeatedly overdose (those who had already received treatment for an overdose twice in the past would not have an ambulance sent to resuscitate them or to receive life-saving medication), as the ‘the financial survivability’ of their city is prioritised over human life [105] (p. 1) in a society that prioritises profit over people [106]. Here, we see the violence and barbarism of biopower [35] and how the irrational practices of the addict, rather than how the nature of consumerist societies, attract denunciation, yet these act as cause, effect and solution to the opioid crisis whilst the systemic harm arising from consumer capitalism and its circuits of consumption is hidden, normalised and individualised [32]. Despite offering a solution to our problems, and acting as a phoenix for some, drug treatments alongside addicts and their representation are merely semblances that keep the neoliberal fantasy alive in a society where symbolism dominates and reality is fetishistically disavowed, as fantasy structures our social reality [54].

Even ‘harm reduction measures’ are not intended to protect the addict. Whether we are talking about the implementation of needle exchanges in the UK during the 1980s heroin epidemic or today’s attempts to equip police officers in America with naloxone to treat overdoses, harm reduction has rarely been implemented to protect and reduce harm to the drug user—addict—as their life does not matter. Instead, these initiatives are implemented to protect real citizens from the addicted other; whether it be the spread of HIV/AIDS into the general population [107] or to protect police officers from fatal exposure to opioids [108]. Addicts threaten to pollute the majority/non-addicts as the lives on non-addicts seem to matter more than that of the addict. In fact, any life matters more than the addict. But does it? In reality, ‘the true problem is not so much the fragile status of the excluded, but rather the fact that, at the most elementary level, we are *all* excluded in the sense that our most elementary, zero position is that of being an object of biopolitics, so much so that political and citizenship rights are granted us only as a secondary gesture, in accordance with strategic of biopolitical considerations’ [39] (p. 124–125, emphasis in original). Neoliberal biopolitics emphasises individual responsibility for health, which is also reflected in drug treatment, as addiction is framed as a personal failing. Here, drug treatment becomes a way to teach self-regulation/self-correction; it fuses/combines the medicalisation and criminalisation of drug treatment and the dual role of coercion and care in biopolitical drug treatment. Like other products in the contemporary marketplace, drug treatment is a sublime object of ideology, which promises redemption from the stigmatised identity of addiction, but the addict is forever cheated [54]. Instead, stigma acts as a barrier to treatment and recovery [3,4] and negatively impacts healthcare professionals’ attitudes to addicts/SUDs, so even if they do access treatment, these stereotypical attitudes result in suboptimal healthcare and poorer treatment outcomes [94].

## 8. Conclusions

Synthesising the work of Fisher and Žižek with Bauman’s concept of a failed wasted life and Girard’s theories of mimetic desire and the generative scapegoat, the preceding discussion has provided a powerful and novel framework for understanding addiction and the addict that moves beyond mono-causal, often individual explanations, to show how capitalist ideology is deceptive and contradictory as it tries to convince us that scapegoated groups of people like drug addicts are responsible for society’s structural problems, ignoring the societal origins of addiction, whilst also disavowing the harm and violence arising from the system itself—a system that has scapegoated certain substances and their users, let them die in fact, to prioritise the exigencies of capitalism (e.g., productivity) and reduce the competition for substances available from the capitalist marketplace.

The proposed theoretical framework not only proffers a more holistic approach to explaining and understanding addiction, but offers a valuable contribution to knowledge by providing a unique perspective on how societal mechanisms and cultural narratives shape the perception and management of drug addicts and addiction. Drawing on Girard’s theory, which revolves around the concepts of mimetic desire, violence, and the scapegoat mechanism, can shed light on the dynamics of stigmatisation, the societal responses to drug addiction (e.g., criminalisation) and illustrate how it detrimentally impacts the health, wellbeing, and recovery of these individuals, as the addict—Muselmann—is a symptom of capitalism. Illustrating the role of broader socio-economic structures in the development and perpetuation of addiction challenges the purely individualistic and biomedical models that have become increasingly dominant (e.g., addiction as a brain disease), as the individualisation of health problems is a reflection of neoliberal ideology [39,109].

Instead, recognising addiction as a societal issue rather than an individual failing can foster more compassionate and effective treatment responses that prioritise the health and wellbeing of the addict over those of the non-addict. As advocated by Link and Phelan [110], only by moving away from individual blame to a broader consideration of the societal and cultural factors underpinning addiction can more compassionate/empathetic, comprehensive and humane approaches to addiction treatment and policy be developed. Recognising addicts as fatalities of societal dynamics rather than solely responsible for their condition can lead to more effective interventions/treatment. The proposed framework facilitates a deeper holistic understanding of the processes that lead to the stigmatisation (and marginalisation) of addicts that is needed globally. This not only applies to the stigmatisation of addicts, but provides a better understanding of stigma more widely, which has been shown to have a negative impact on the health of the population for majority and minority groups [29].

While the public continue to see addiction as a personal moral failing, they are more likely to oppose policies aimed to help addicts recover and remain unconvinced about the effectiveness of drug treatment [2]. Re-framing addiction as an (accepted) societal phenomenon, rather than as a moral failing, criminal and/or personal weakness, can lead to profound changes at the policy level by fostering a shift in how governments, institutions, and communities see and approach addiction. Only by framing addiction this way can stigma be reduced (e.g., via education and public health campaigns) and policies addressing social determinants of health (e.g., housing, education), that focus on treatment and harm reduction rather than punishment be implemented, and healthcare systems and harm reduction initiatives receive more funding over law enforcement and imprisonment. Re-framing addiction as an accepted societal phenomenon can lead to policies that address addiction holistically and prioritise compassion, health, and social equity over criminalisation, punishment, discrimination, and inequality to create more effective and humane approaches to managing addiction. Consequently, moving away from a reductionist view, this unique framework proffers a more nuanced analysis of the addict in contemporary society, which will have significant health benefits not only by helping to understand barriers to treatment and harm reduction initiatives, but also stigmatisation and scapegoating, which can lead to worsening mental and physical health, social isolation, and negative effects that ultimately perpetuate drug use and the cycle of addiction.

The novel tripartite framework emphasises how multiple forces seek to emphasise, utilise, and continue the stigmatising framing of the drug addict as a tool to maintain the dominant social order. Resultantly, it has significant implications for drug policy and practice. Firstly, it indicates the need to live with rather than without the drug addict, aligning with Buchanan’s [85] calls for an overhaul of drug prohibition and a move towards a regulatory system for all legal/illegal drugs formulated around principles of human rights to all, including drug addicts. Secondly, it indicates that addiction is part of our consumerist social fabric and, in doing so, frames this as behaviour which conforms to rather than diverts social norms, which implies that drug services should not be isolated but incorporated into mainstream health services, negating certain elements of isolation and exclusion. Thirdly, it reminds us that most drug addicts achieve recovery without the need to access treatment services. This is not to say that such services are not critical for some, but treatment is not the only way of addressing one’s addiction, and for some it can actually be a demoralising and ineffective intervention. Fourthly, it highlights that the burgeoning drug treatment market is often grounded in mythical promises and shrouded in a luxurious and overstated cloak of effectiveness, thus fitting notions of conspicuous consumption rather than a robust evidence base. Whilst consumer capitalism is here to stay, as are its damaging processes, this framework at least provides a blueprint to how we might work within such circuits to lessen stigmatisation, empower and recognise the right to be a drug addict, and provide less isolating and more impactful support to those who are addicted to drugs and wish to access such resources. Until then, we will continue to inflict harm on drug addicts, which is probably more harmful than the drugs they are addicted to.

The addict is a social contradiction, which reflects the ambiguities inherent in consumer capitalism and its erratic excess. Addicts are the Muselmann, serving as a stark reminder to us all of the brutal realities of biopolitical power. Reduced to Agambian bare life, the Muselmann parallels how scapegoated individuals or groups are stripped of their humanity and seen merely as the embodiment of societal ills. The addict is the monstrous other. The addict is the ideological scapegoat; the symptom that helps to sustain capitalism by directing desires and providing coherence to the otherwise chaotic and excessive nature of capitalist consumption. The marginalised addict is framed as the cause of their own downfall and the destruction of society, which is packaged up and sold back to us as a warning, whilst redemption is framed as achievable via solutions offered by the legitimate marketplace as ‘capitalism offers the promise of belonging with every commodity’ [63] (p. 21). Drug treatment promises redemption via recovery and reintegration. The problem is, many drug addicts were never socially included in the first place, so re-entry into ‘society seems a long way off for many problem drug users’ [85] (p. 396), making it impossible to break through the stigma that has led to their vilification and exclusion. Despite promising redemption from addiction, the road to and during recovery is one littered with solutions that fail to address the wider notions of stigmatisation and structural inequalities that such individuals face, which are disavowed [40].

Consequently, drug addicts are the weird; ‘that which does not belong’, or that which we think does not belong [41] (p. 10). Addicts are also the eerie [41]. Capitalism ambiguously demands enjoyment that is prohibited and addiction contravenes the acceptable and commodified versions of enjoyment tolerated by society, ignoring the restrictions and limitations the symbolic order puts on enjoyment, as unrestrained consumption has become one of the main focuses of today’s biopolitics, as every aspect of life has become commodified and individualised, including health [2,15,39]. Consuming to excess is not their only sin, however; they also seek pleasure without a detour through the symbolic, which implodes their desire for other commodities and thus threatens to halt the desire and self-perpetuation that capitalism relies upon [11]. While many of us are addicted to something, we are addicted to the right things (e.g., wealth and monetary success) and the right substances (e.g., coffee/tea, sugar, caffeine) for now, as consumer capitalism is constantly revolutionising itself and shifting the boundaries.

Therefore, the preceding discussion maps out how consumer capitalism stigmatises and frames addiction as a failure, and while feasting on such failures, it showcases its sovereignty through a veneer of redemption, which allows the failed phoenix to be reborn through responsible consumption bought from the legitimate marketplace. Stigma seems to have become a ‘legitimate public health tool’ and an integral part of many global health campaigns (e.g., smoking, obesity) [111] (p. 7), despite the harms arising from it. Redemption through consuming drug treatment promises to remove the stigma, and ‘it is getting rid of that stigma that now conditions happiness; and happiness…needs to be paid for’ [13] (p. 37). Drug treatment deceitfully promises to alleviate suffering and maximise individual opportunities, but the addict is forever cheated, as what we achieve is never that, as ‘this paradox concerns the functioning of surplus in our libidinal economy’ as we ‘desire the surplus that eludes every object, our very orientation towards pleasure and satisfaction compels us to permanently sacrifice available satisfactions on behalf of satisfactions to come’ [91] (p. 160). In doing so, it demonstrates how failed consumption, the consumption of failure and the penance of the failed are entwined with the contradictory practices of consumerism and its erratic excess, as all constitute sublime objects of ideology that operate to keep the neoliberal fantasy alive [54]. Consequently, addicts are an ideologically constructed fantasy image that is failure, feast and phoenix.
